# MagA expression attenuates iron export activity in undifferentiated multipotent P19 cells

**DOI:** 10.1371/journal.pone.0217842

**Published:** 2019-06-06

**Authors:** Linshan Liu, Kobra Alizadeh, Sarah C. Donnelly, Praveen Dassanayake, Tian Tian Hou, Rebecca McGirr, R. Terry Thompson, Frank S. Prato, Neil Gelman, Lisa Hoffman, Donna E. Goldhawk

**Affiliations:** 1 Imaging, Lawson Health Research Institute, London, Ontario, Canada; 2 Medical Biophysics, Western University, London, Ontario, Canada; 3 Collaborative Graduate Program in Molecular Imaging, Western University, London, Ontario, Canada; 4 Microbiology and Immunology, Western University, London, Ontario, Canada; 5 Medical Imaging, Western University, London, Ontario, Canada; 6 Physics and Astronomy, Western University, London, Ontario, Canada; 7 Anatomy and Cell Biology, Western University, London, Ontario, Canada; Stanford University, UNITED STATES

## Abstract

Magnetic resonance imaging (MRI) is a non-invasive imaging modality used in longitudinal cell tracking. Previous studies suggest that MagA, a putative iron transport protein from magnetotactic bacteria, is a useful gene-based magnetic resonance contrast agent. Hemagglutinin-tagged MagA was stably expressed in undifferentiated embryonic mouse teratocarcinoma, multipotent P19 cells to provide a suitable model for tracking these cells during differentiation. Western blot and immunocytochemistry confirmed the expression and membrane localization of MagA in P19 cells. Surprisingly, elemental iron analysis using inductively-coupled plasma mass spectrometry revealed significant iron uptake in both parental and MagA-expressing P19 cells, cultured in the presence of iron-supplemented medium. Withdrawal of this extracellular iron supplement revealed unexpected iron export activity in P19 cells, which MagA expression attenuated. The influence of iron supplementation on parental and MagA-expressing cells was not reflected by longitudinal relaxation rates. Measurement of transverse relaxation rates (*R*_2_* and *R*_*2*_) reflected changes in total cellular iron content but did not clearly distinguish MagA-expressing cells from the parental cell type, despite significant differences in the uptake and retention of total cellular iron. Unlike other cell types, the reversible component *R*_*2*_*′* (*R*_2_* ‒ *R*_*2*_) provided only a moderately strong correlation to amount of cellular iron, normalized to amount of protein. This is the first report to characterize MagA expression in a previously unrecognized iron exporting cell type. The interplay between contrast gene expression and systemic iron metabolism substantiates the potential for diverting cellular iron toward the formation of a novel iron compartment, however rudimentary when using a single magnetotactic bacterial gene expression system like m*agA*. Since relatively few mammalian cells export iron, the P19 cell line provides a tractable model of ferroportin activity, suitable for magnetic resonance analysis of key iron-handling activities and their influence on gene-based MRI contrast.

## Introduction

Molecular imaging has been used in the characterization and measurement of biological processes [1] by exploiting molecular probes as contrast agents for imaging modalities like X-ray computed tomography (CT), positron emission tomography (PET) and magnetic resonance imaging (MRI) [2]. For longitudinal cell tracking by MRI, a few techniques for tracking magnetically labeled cells have been proposed, including gene-based approaches [3]. There are numerous advantages of noninvasive imaging using gene-based contrast. Since the label remains with the cell throughout its life cycle, individuals may be imaged repeatedly to track development or progress of disease. Such *in vivo* imaging may also include reporter gene expression of specific transcription factor (TF) activity, thereby identifying the onset of differentiation; determining the sequence of TF expression; establishing the temporal and spatial regulation of TF activity; and clarifying the functional ability of TF protein to drive expression of downstream genes.

Previous studies suggest that MagA, a putative iron transport protein found in magnetotactic bacteria, can be used as an endogenous contrast agent in mammalian cells for MRI [4–7]. These reports indicate that MagA is involved in increasing cellular iron content, as confirmed by magnetic resonance (MR) relaxation rates and elemental analysis, without introducing cytotoxicity. While many reports of MagA expression involve cancer cell models [8], relatively few explore stem cell models [4]. Rectifying this deficiency would open up new options for addressing current challenges in stem cell therapy. There remains a need to understand the fate of transplanted cells, their localization in target tissues, degree of functionality and therapeutic window. Many advantages of MRI over other *in vivo* imaging techniques are ideal for this type of molecular imaging. This includes the use of nonionizing radiation for repetitive imaging; exceptional image resolution (1 mm^3^ isotropic on clinical scanners and approximately 0.1 mm^3^ on preclinical scanners); as well as versatile image acquisition for multiparametric imaging. In addition, with gene-based contrast and the advent of hybrid imaging platforms, like PET/MRI, multiple activities could be tracked in a single imaging session with complete registration [9, 10].

In the present study, we provide the first report of MagA expression in the P19 mouse embryonal teratocarcinoma cell line. This multipotent cell type is capable of differentiation down the three cell lineages and provides an easily cultured model of stem cell behavior. In undifferentiated cells, we used a hemagglutinin (HA) tag to verify MagA protein expression and localization. We examined the response of parental and MagA-expressing P19 cells to culture in the presence and absence of an extracellular iron supplement, measuring total cellular iron content by inductively-coupled plasma mass spectrometry (ICP-MS). In addition, we used a previously developed cell phantom to measure the relaxation rates of parental P19 cells and those expressing MagA using 3 Tesla (3T) MRI [6]. Whereas we expected that MagA expression would increase cellular iron and MR contrast as reported for other cell types [4–7], this study revealed surprising iron handling activity in the parental P19 cells, including iron export, which MagA expression attenuated.

## Materials and methods

### MagA expression in P19 cells

#### Reagents

Unless otherwise noted, molecular and cell biology reagents were purchased from Life Technologies (Burlington, Canada). Ferric nitrate and buffer salts were purchased from Sigma-Aldrich (Oakville, Canada).

#### Vector construct

The epitope-tagged gene, *magA-HA*, was inserted into pcDNA3.1Zeo(+), under the control of the cytomegalovirus constitutive promoter. Briefly, using customized primers to incorporate the HA sequence (underlined in Table 1) and a published protocol [5], *MagA* was cloned by PCR from *Magnetospirillum magneticum* sp. AMB-1 (ATCC # 700264, Burlington, Canada). The resultant *MagA-HA* PCR fragment was sub-cloned into pCR2.1-TOPO and shuttled into pcDNA3.1Zeo(+) at Kpn I/Bam H1.

**Table 1:** Primer sequences used to clone *magA-HA*.

Primer	Sequence (5' to 3')
Forward	GGTACCGCCACCATGGAACTGCATCATCCCGAACTGACCTATGCCGCCATCG
Reverse**^**	CCGAGACCTTAACTTAAGATAGGCATACTACACGGCCTAATACGCATTCCTAGGCG

**^** Sequence encoding HA is underlined.

#### Cells

Mouse multipotent teratocarcinoma cells (P19, ATCC # CRL-1825) were cultured in α-minimum essential medium (αMEM)/10% fetal bovine serum (v/v) and maintained under standard cell culture conditions at 37°C with 5% CO2. At approximately 80% confluence, cells were routinely passaged at a 1/10 dilution using 0.25% Trypsin/0.91 mM EDTA.

#### Transfection

Transfection was performed using Lipofectamine 2000 according to the manufacturer’s instructions. Cells were 70–80% confluent on the day before transfection and replated approximately 24 hours after transfection at a 1/20 dilution. Selection began 24 hours post-transfection using 200 μg Zeocin/mL medium. After approximately two weeks under selection, distinct colonies of P19 cells appeared on the plate. Several of these colonies were randomly selected and individually replated in 6–well plates for further amplification on 100 mm^2^ dishes. At confluence, clonal lines were placed in cryostorage until examined by Western blot for MagA expression, as described below.

#### Iron supplementation

To examine MagA activity, cells were cultured in the presence or absence of iron-supplemented medium, containing 250 μM ferric nitrate. This level of extracellular iron approximates the total bound and unbound iron pool present in the blood [5, 7, 11]. Following 7 days of iron supplementation, select plates were washed twice with phosphate buffered saline (PBS) pH 7.4 (137 mM NaCl/2.7 mM KCl/10 mM Na_2_HPO_4_) and returned to non-supplemented medium for an additional 24 hours of culture. In parental P19 cells, iron uptake was also examined using 25 μM ferric nitrate and 2 days of iron-supplemented culture. At harvest, all cells were washed twice with PBS and either prepared for MRI (described below) or collected for protein analysis. The latter cell samples were scraped off the plate in 1 mL ice-cold 50 mM Tris-HCl pH 8/5 mM EDTA/150 μL Complete Mini protease inhibitor cocktail (Roche Diagnostic Systems, Laval, Canada) and lysed by sonication. Protein concentration was quantified using the BCA Protein Assay Kit (ThermoFisher Scientific, Mississauga, Canada) [12].

#### Reverse transcription polymerase chain reaction (RT-PCR)

RT-PCR was performed using 30 cycles and an annealing temperature of 60°C. The reaction contained 4 μL cDNA reverse transcribed from 1 μg total RNA, which was purified from P19 cells cultured in the presence (+Fe) and absence (-Fe) of iron supplementation, as described above. The fragment amplified by mouse transferrin receptor primers (forward 5' CTCGGCAAGTAGATGGAGATA 3' and reverse 5' ATGGAGTTCAACTTCTCTGA 3') is 337 bp and was visualized on a 1% agarose gel with ethidium bromide. The control PCR used water instead of cDNA template.

#### Western blot

To confirm expression, cellular protein from clonal lines of MagA-HA-expressing P19 cells were separated by sodium dodecyl sulfate polyacrylamide gel electrophoresis (SDS PAGE, 40 μg/lane) on a precast 4–12% gradient gel (Life Technologies) and transferred to a nitrocellulose membrane using the Original iBlot Gel Transfer Device (Life Technologies). Blots were blocked with 5% Blotto/Tris buffered saline (TBS, 50 mM Tris-HCl pH 7.6/0.9% NaCl)/0.1% Tween 20 (TBST) for one hour, followed by overnight incubation at 4°C in primary monoclonal mouse α-HA (1/1000 in 1% Blotto/TBST). The following day, membranes were washed four times in TBST and incubated for 1 hour with secondary horseradish peroxidase (HRP)-conjugated goat α-mouse immunoglobulin (Ig, Sigma-Aldrich, Oakville, Canada), diluting 1/5000 in 1% Blotto/TBS. Immunoblots were subsequently washed 3 times with TBST and once with TBS prior to development with a chemiluminescent substrate (Super Signal West Pico, ThermoFisher Scientific). Chemiluminescence was captured using GeneSnap 7.12 Software (Cambridge, England) while exposed 5 min in the Chemigenius Gel Doc (Syngene).

#### Immunocytochemistry (ICC)

Sterile glass coverslips were placed in 6-well plates and rinsed with PBS prior to seeding approximately 10^6^ MagA-HA-expressing P19 cells/well. At 70% confluence, cells were washed with PBS; fixed with 2% paraformaldehyde/PBS for 30 min; and permeabilized with 0.25% Triton X-100/PBS for five min. After washing three more times with PBS, cells were incubated with blocking buffer (10% goat serum/1% bovine serum albumin (BSA)/PBS) for one hour. Cells were subsequently incubated overnight at 4°C with goat α-HA (Abcam, Toronto, Canada, 1/100 dilution in blocking buffer). Golgi Apparatus and plasma membrane were visualized using mouse α-Golgi-associated protein p115 (Transduction Laboratories, Lexington, KY, 1/50 in blocking buffer) and Alexa Fluor 594-conjugated wheat germ agglutinin (WGA, Life Technologies, 5 μg/mL in blocking buffer), respectively. The secondary antibodies used were Alexa Fluor 488-conjugated donkey α-goat Ig and Alexa Fluor 594-conjugated donkey α-mouse Ig (Life Technologies), both at a 1/500 dilution. After 2 to 4 hours of secondary antibody incubation, cover slips were mounted on glass slides using ProLong Gold Antifade Reagent (Life Technologies). Cells were then visualized on an Olympus IX81 wide field fluorescence microscope. Image acquisition was carried out using In Vivo software. Ten optical sections per cell were collected in 0.2 μm steps covering the z-axis field, using a 60× oil immersion objective. Cell images were processed using a 3-dimensional blind deconvolution algorithm provided by Image-Pro Plus software (Media Cybernetics, Rockville, MD).

#### Trace element analysis

Samples were sent to the Analytical Services Laboratory of Surface Science Western (Western University, London, Canada) for trace element analysis of iron and zinc using ICP-MS. Samples were prepared from cells cultured as described above. At harvest, cells were prepared as for SDS PAGE. Cellular iron and zinc content were normalized to total cellular protein. For each individual sample group, mean and standard error of the mean (SEM) were calculated in Excel, version 14.3.8.

### MRI of MagA-expressing cells

#### Phantom preparation

**Fig 1:**
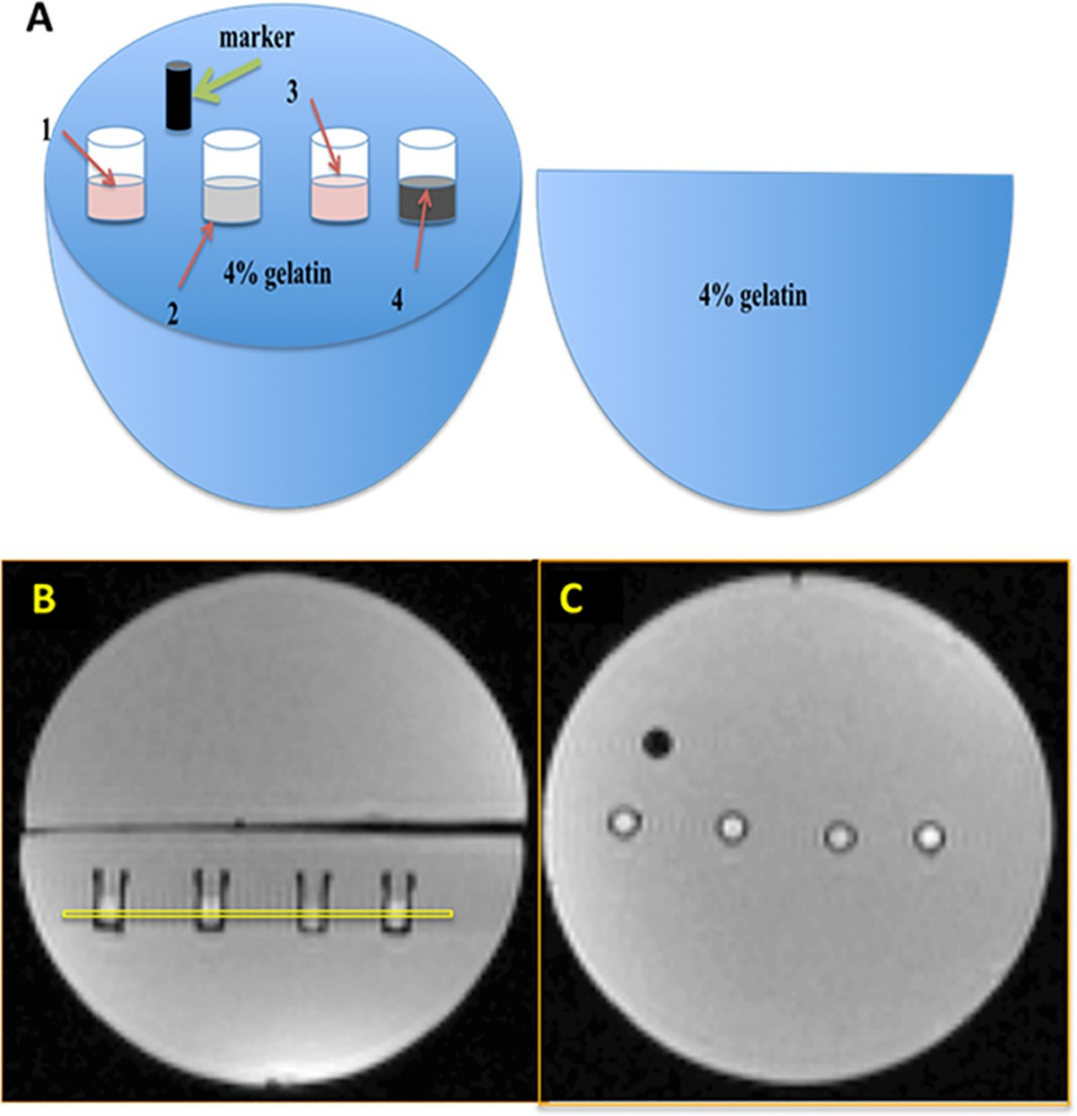
MRI cell phantom. The diagram (A) and MR localizer images (B, C) indicate the sample layout within a plastic 9-cm spherical mold, filled with 4% gelatin/PBS. Four different samples, prepared from parental and MagA-expressing cells cultured in the presence (+Fe) and absence of iron supplementation, were inlaid in one hemisphere and arranged from left to right as follows: P19 (1), P19 + Fe (2), MagA (3), MagA + Fe (4). A plastic marker (black) indicates orientation of the samples. Images were acquired by 3T MRI [6]. In the sagittal view of the phantom (B), a yellow box indicates the 1.5 mm slice selected for image acquisition. A cross sectional view of the phantom (C) shows the alignment of sample wells.

Fig 1 depicts the spherical MRI cell phantom used for measurement of relaxation rates. Approximately 40–50 million cells were placed in a custom-made well, fabricated from an NMR-compatible material (Ultem, Lawson Imaging Prototype Lab). The dimensions of each cylindrical well are: inner diameter 4 mm and height 10 mm. Samples were centrifuged 5 min at 400 × g to create a compact layer of cells within each well. These cell pellets were overlaid with 1% gelatin (porcine type A, Sigma-Aldrich)/PBS and embedded in one hemisphere of a 9 cm spherical phantom filled with 4% gelatin/PBS. A spherical-shaped phantom was used to minimize macroscopic magnetic field inhomogeneity which would interfere with accurate R2′ measurement. Samples consisted of either parental or MagA-expressing cells, cultured under different conditions of iron supplementation: without extra iron supplementation, with iron supplementation (250 μM ferric nitrate), and withdrawal of iron supplementation after 7 days of continuous iron supplementation. To form the spherical gelatin phantom, the empty hemisphere was filled with 4% gelatin/PBS and placed on top of the half containing cell samples. Using a layer of parafilm, air was excluded in order to avoid susceptibility artifacts at the interface [6].

#### Image acquisition

The spherical phantom was placed in a 15-channel knee radiofrequency (RF) coil and scanned on a 3T mMR Biograph (Siemens AG, Erlangen, Germany) using previously described sequences [6]. To acquire T1-weighted images, inversion recovery spin echo sequences were used. Imaging parameters were as follows: echo time (TE) = 13 ms; repetition time (TR) = 4000 ms; six inversion times (TI) = 22, 200, 500, 1000, 2000, 3900 ms; total scanning time approximately 39 min. To acquire T2-weighted images, a single echo spin echo sequence and the following imaging parameters were employed: TE = 13, 30, 40, 60, 80, 100, 150, 200, 300 ms; TR was fixed at 1000 ms (TR ‒ TE); total scanning time approximately 61 min. To acquire T2*-weighted images, a multi echo gradient echo sequence was used: TE = 6.12, 14.64, 23.16, 31.68, 40.2, 50, 60, 70, 79.9 ms; TR = 200 ms; flip angle = 60^o^; total scanning time approximately 25 min. For all MR images, the field of view was 120×120 mm. For T1-weighted images, the volume of the voxels was 1.5×0.9×0.9 mm^3^ and matrix size was 128×128. For T2- and T2*-weighted images, the voxel size was 1.5×0.6×0.6 mm^3^ and matrix size was 192×192. Slice thickness was 1.5 mm and selected as shown in Fig 1B. Slices were oriented perpendicular to the sample wells to obtain a cross section through the cell layer and avoid voxels from the bottom of the well and the top gelatin layer. Manual selection of the region of interest (ROI) was performed using a graphic user interface based on Matlab 7.9.0 (R2010b) [6] and included as many voxels as possible while excluding those closest to the wall of the well (Fig 1C). There were approximately 20 voxels in each ROI and average signal intensity of the ROI for each time point and relaxation rate (R2 = 1/T2; R2* = 1/T2* and R1 = 1/T1) was determined with least-squares curve fitting (SigmaPlot 10.0, Systat Software, Germany) of the mean ROI signal.

#### Calculation of relaxation rates

R1 decay curves were obtained using an inversion recovery pulse sequence. R1 was determined with least-squares curve fitting of the mean ROI signals using Eq 1.

$$S(TI)=|S_{0}\cdot ({1-2\cdot e}^{-TI\cdot R1}+e^{-TR\cdot R1})|\cdot e^{-TE/T2}$$ 1

R2* decay curves (average signal intensity over all TE) were obtained from the data acquired using multi-echo spin echo sequences. R2 curves were obtained from single echo spin echo pulse sequences. Sigmaplot 10.0 was used to fit R2 and R2* curves with a single exponential decay equation. A two-parameter model was tested using the following equations.

$$S(TE)=S_{0}e^{-TE\cdot R2}$$ 2

$$S(TE)=S_{0}e^{-TE\cdot {R2}^{*}}$$ 3

Once R2* and R2 were determined, R2′ was calculated from their difference (R2′ = R2* ‒ R2). For each individual sample group, mean and standard error of the mean (SEM) of relaxation rates (R1, R2*, R2 and R2′) were calculated in Excel.

#### Statistical analysis

Samples were assigned to six groups: 1) parental P19 cells (P); 2) MagA-expressing P19 (M); 3) iron-supplemented parental cells (P+Fe); 4) iron-supplemented, MagA-expressing P19 cells (M+Fe); 5) P+Fe cultured an additional 24 hours in non-supplemented medium (P 24h-Fe); and 6) M+Fe cultured an additional 24 hours in non-supplemented medium (M 24h-Fe). Two-way analysis of variance (ANOVA) was used to assess main effects and interaction between variables. One-way ANOVA was used to compare interaction of iron treatment(s) within a given cell type. Analyses were performed with SPSS version 20.0. Student’s t-tests were used to evaluate significant differences between parental and MagA-expressing P19 cells for each condition of iron supplementation. Linear regression was tested with R2′ as the dependent variable and iron concentration as the independent variable. P < 0.05 was the threshold of statistical significance. Error bars indicate standard error of the mean (SEM).

## Results

### Generation of a P19 cell line stably-expressing MagA

**Fig 2:**
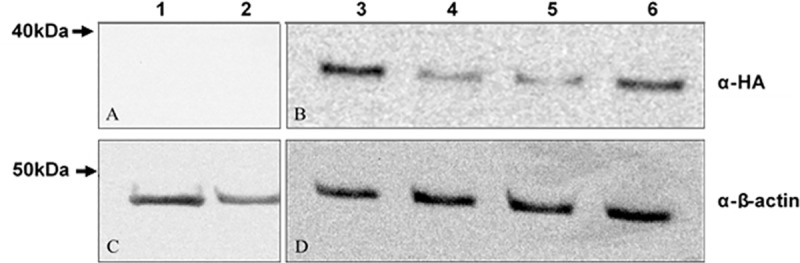
Western blots confirm expression of MagA in P19 cells. Lanes 1 and 2 contain protein extracted from untransfected parental P19 cells while lanes 3–6 contain protein from MagA-HA-expressing cells. Panels A and B were probed with antibody against HA. Panels C and D were probed with an antibody against the loading control, β-actin. Approximate M.W. is indicated on the left.

Total protein from both untransfected parental P19 and MagA-HA-expressing cells was analyzed by Western blot. As shown in Fig 2A, parental cells (lanes 1–2) showed no α-HA immunostaining (refer to S1 Fig). In contrast, varying degrees of HA-tagged protein are detected as a single band in transfected cells, stably expressing MagA-HA (lanes 3–6, Fig 2B). The predicted molecular weight (M.W.) of MagA-HA is 46.8K while the apparent M.W. of α-HA bands is approximately 35K, similar to a prior report [4]. In each lane, the β-actin control appeared as a single uniform band at approximately 40K (Fig 2C and 2D). After confirming the presence of HA-tagged protein (Fig 2B), the highly expressing clone in lane 3 was selected for use in all subsequent experiments.

### Subcellular localization of MagA

**Fig 3:**
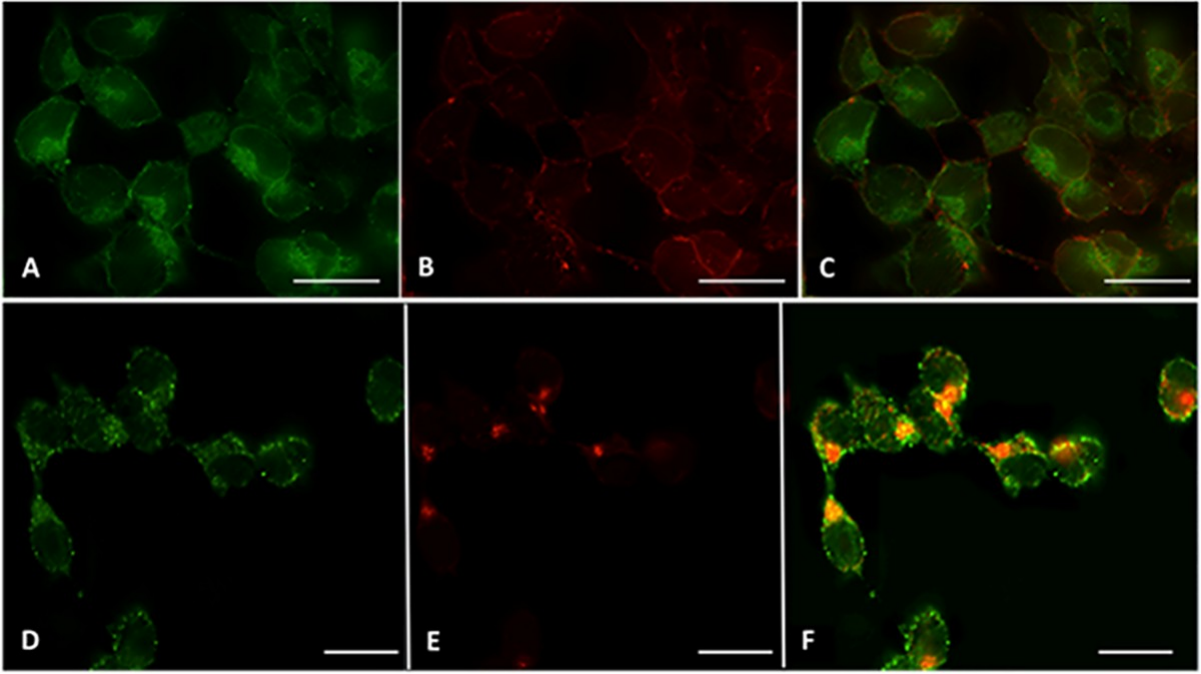
MagA is mainly localized in the intracellular compartment. P19 cells stably expressing MagA-HA were sequentially probed first with primary goat α-HA and secondary Alexa Fluor 488 conjugated-donkey α-goat Ig (A, D, green fluorescence) and then with either Alexa Fluor 594-conjugated WGA (B, red fluorescence) or primary mouse α-p115 and secondary Alexa Fluor 594-conjugated donkey α-mouse Ig (E, red fluorescence). Merging panels A and B revealed only mild yellow fluorescence (C) at the plasma membrane. However, merging panels D and E revealed clear yellow fluorescence (F) wherever MagA-HA and p115 co-localized in the Golgi Apparatus. Scale bar = 15 μm.

Using ICC and an HA antibody, we examined the cellular localization of MagA in P19 cells. As shown in Fig 3A and 3D, MagA expression appears somewhat punctate both within the cell and at its periphery. Counterstaining the plasma membrane with fluorescently-conjugated WGA (Fig 3B) indicates relatively little colocalization at the plasma membrane (Fig 3C). To address the intracellular localization of MagA, cells were immunostained with an antibody specific to the cis-Golgi Apparatus, membrane-associated protein p115 (Fig 3E). The overlay of HA and p115 staining (Fig 3F) reveals intracellular yellow fluorescence consistent with co-localization of MagA and cis-Golgi Apparatus membrane.

### Analysis of cellular iron in MagA-expressing P19 cells

**Fig 4:**
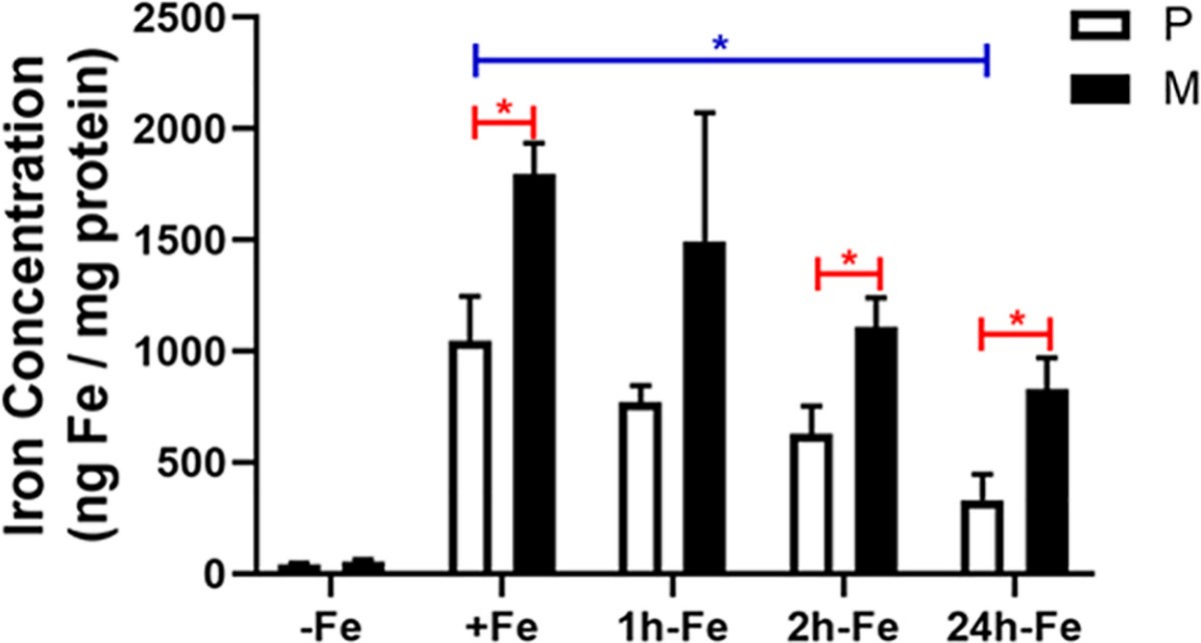
Elemental analysis of iron in parental and MagA-expressing P19 cells and time course of iron export. Total cellular iron content was analyzed by ICP-MS and normalized to total cellular protein. Parental P19 cells (P, white bars) and those expressing MagA (M, black bars) were cultured at least 7 days in the presence (+Fe) and absence (-Fe) of iron supplementation (250 μM ferric nitrate∕medium) prior to withdrawal of iron supplement and culture for an additional 1 (1h-Fe), 2 (2h-Fe) and 24 (24h-Fe) hours. Total cellular iron content was significantly higher (p < 0.001) in both iron-supplemented cell types (+Fe) compared to the non-supplemented samples (-Fe) and remained higher for one hour after iron withdrawal (p < 0.01). Iron content in MagA-expressing cells was higher than untransfected cells after iron supplementation (+Fe, red *) and from 2h-Fe to 24h-Fe. Total cellular iron content decreased significantly in parental cells over 24 hours (blue *) but not in MagA-expressing cells. Error bars are ± SEM (* p < 0.05). For -Fe, n = 4–8; for +Fe, n = 5–7; for all other samples n = 3.

The total cellular iron content of MagA-expressing cells was examined using ICP-MS and compared to the parental control. As shown in Fig 4, elemental iron in both parental and MagA-expressing cells is significantly increased after culture in iron-supplemented medium containing 250 μM ferric nitrate (P vs. P+Fe, p < 0.001; M vs. M+Fe, p < 0.001). After iron supplementation for 7 days, parental and MagA-expressing P19 cells contain approximately 1047 ± 200 and 1797 ± 138 ng Fe/mg protein, respectively; whereas, in unsupplemented culture, parental and MagA-expressing P19 cells contain approximately 30-fold less iron: 41.3 ± 5.9 and 53.9 ± 10.4 ng Fe/mg protein, respectively. These data show that MagA expression does not cause any significant change in cellular iron content in the absence of an extracellular iron supplement, consistent with previous findings in MDA-MB-435 cells [6]. After 7 days of iron supplementation, total cellular iron content was significantly greater in MagA-expressing P19 cells compared to the parental control.

To further delineate the iron handling activities of P19 cells, their ability to retain iron after the withdrawal of extracellular supplement was examined (Fig 4). Accordingly, after 7 days of iron supplementation, both parental and MagA-expressing cells were returned to unsupplemented medium for an additional 24 hours of culture. Under these conditions, a sharp decline in iron level was observed in both the parental and MagA-expressing cells over 1–2 hours, which continued to diminish over 24 hours (S2 Fig). However, total cellular iron content of MagA-expressing cells remained significantly higher than the parental control and was maintained over 24 hours at a higher level than in parental P19 cells.

Altogether, these data show that untransfected and MagA-expressing P19 cells have different capacities to incorporate significant amounts of iron from an extracellular supplement and different abilities to retain this iron. The parental P19 cell type demonstrates a substantial iron export activity (+Fe vs. 24h-Fe, p < 0.05) which is attenuated by MagA expression. Even 24 hours after the withdrawal of iron supplement, MagA-expressing cells retain significantly more iron (830 ± 141 ng Fe/mg protein), approximately 2.5-fold higher than the parental control (330 ± 117 ng Fe/mg protein, p < 0.05).

**Fig 5:**
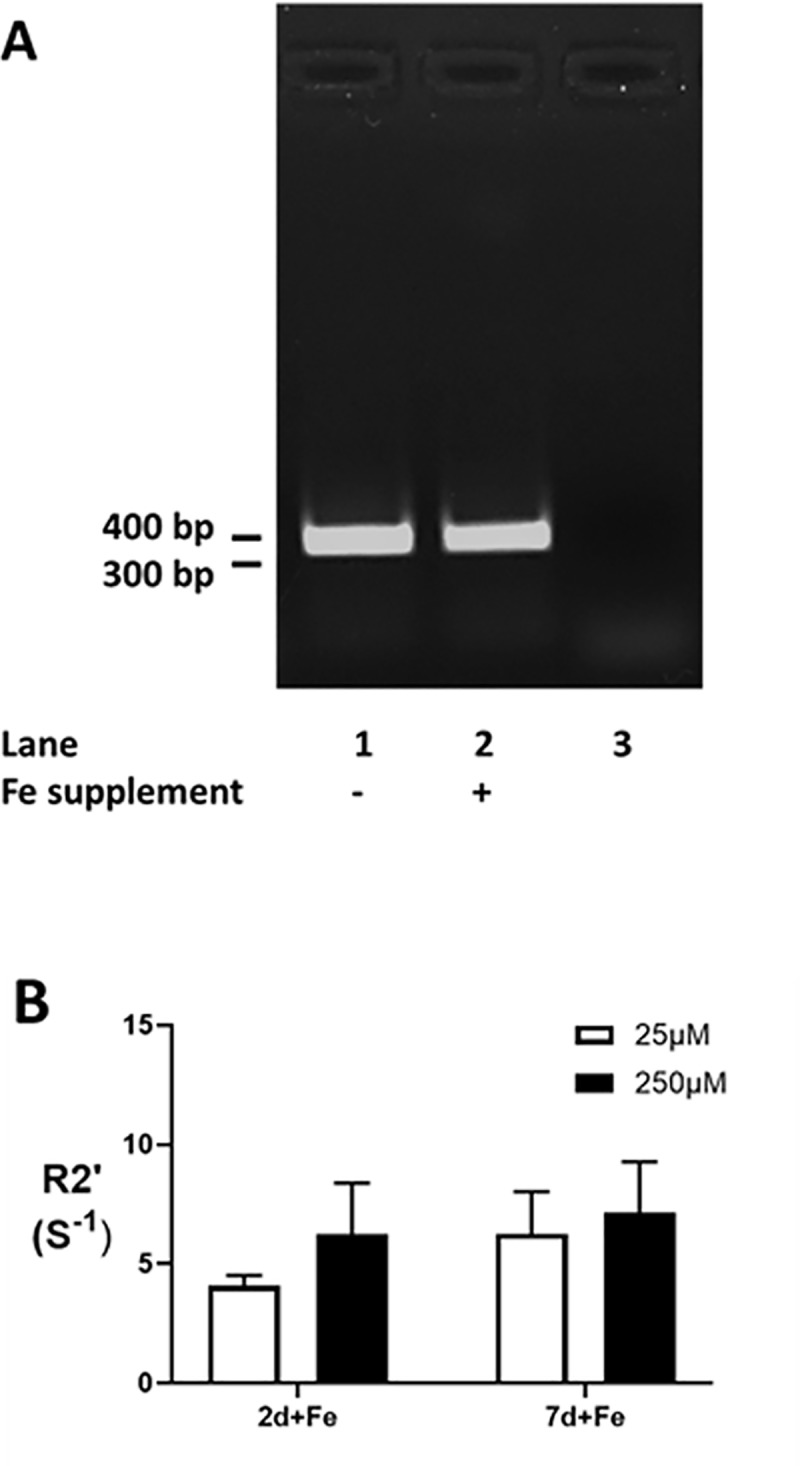
Influence of time and concentration on iron uptake in P19 cells. Transferrin receptor expression in parental P19 cells, cultured in the presence (P+Fe) and absence (P-Fe) of iron supplementation, was examined by RT-PCR (A) and indicates little or no change in response to extracellular iron. Lane 3 shows the control PCR where cDNA template was replaced with water. The influence of iron concentration in the extracellular supplement was also examined (B). Despite a 10-fold difference in iron supplement, 25 μM ferric nitrate (white bars) versus 250 μM (black bars), there was no significant difference in the iron-specific R2′ transverse relaxation rate [13, 14] after either 2 (2d+Fe, n = 3–4) or 7 (7d+Fe, n = 4–9) days in culture.

Characterization of iron uptake in parental P19 cells shows that transferrin receptor expression is not strongly influenced by 7 days in iron-supplemented culture (Fig 5A). This cell type is programmed for iron import. In addition, there is no significant difference in the iron-specific, R2′ transverse relaxivity [13, 14] when iron supplement is reduced from 250 to 25 μM or when the time in iron-supplemented culture is reduced from 7 to 2 days (Fig 5B).

### MRI of MagA-expressing cells

**Fig 6:**
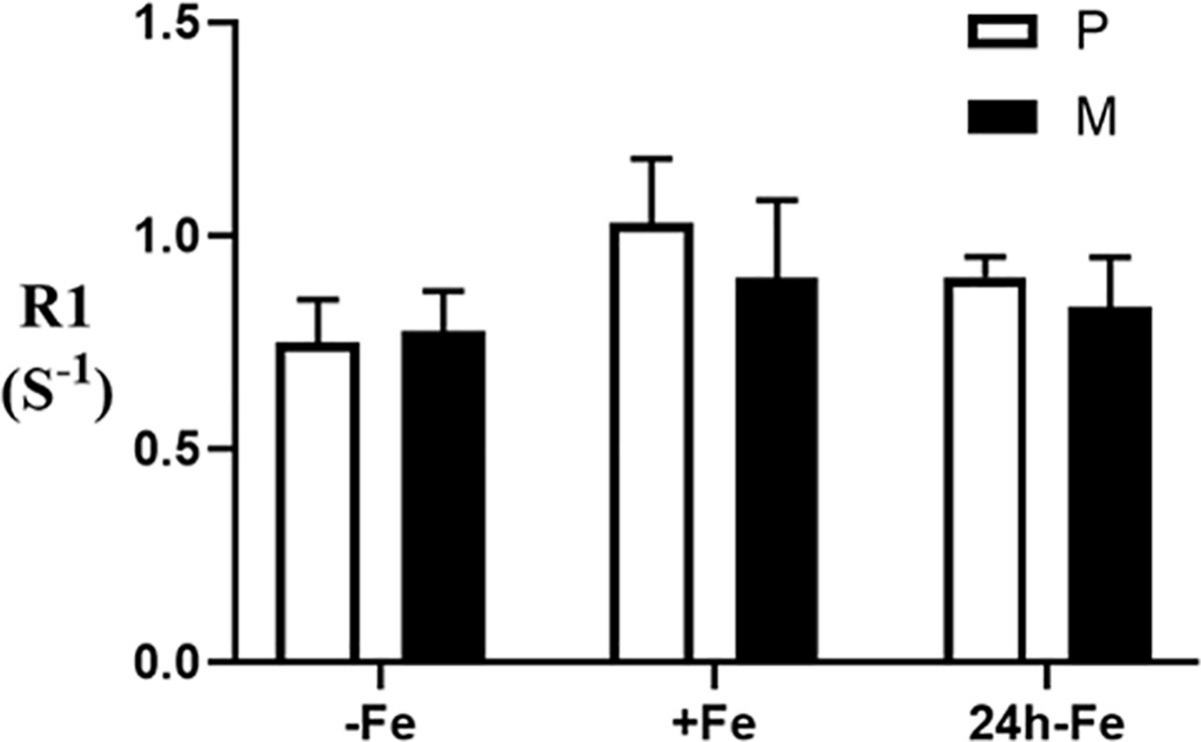
Influence of iron supplementation and MagA expression on longitudinal relaxivity in P19 cells. Longitudinal relaxation rate (R1) was determined in parental cells (P, white bars) and MagA-expressing cells (M, black bars) cultured in the absence of iron supplementation (-Fe), in medium containing 250 μM ferric nitrate for 7 days (+Fe) and after withdrawal of iron from +Fe samples for 24 hours (24h-Fe). Cells were mounted in gelatin phantoms and scanned at 3T as previously described [6]. There is no significant difference between cell types either within or between any given culture condition. Error bars are ± SEM; for -Fe samples, n = 4; for +Fe samples, n = 4–7; for 24h-Fe samples n = 3–7.

To relate changes in cellular iron content with the associated MR signal, phantoms containing cell pellets from parental and MagA-expressing P19 samples were scanned at 3T. Fig 6 shows the mean values of the longitudinal relaxation rates in each cell type under three different culture conditions: -Fe, no iron supplementation; +Fe, iron supplementation for at least one week; 24h-Fe, at least a week of iron supplementation (+Fe) followed by an additional 24 hours of culture in non-supplemented medium. No significant differences in R1 measurements, which ranged between 0.7–1.1 s^-1^, were observed between samples (n = 29 in total).

**Fig 7:**
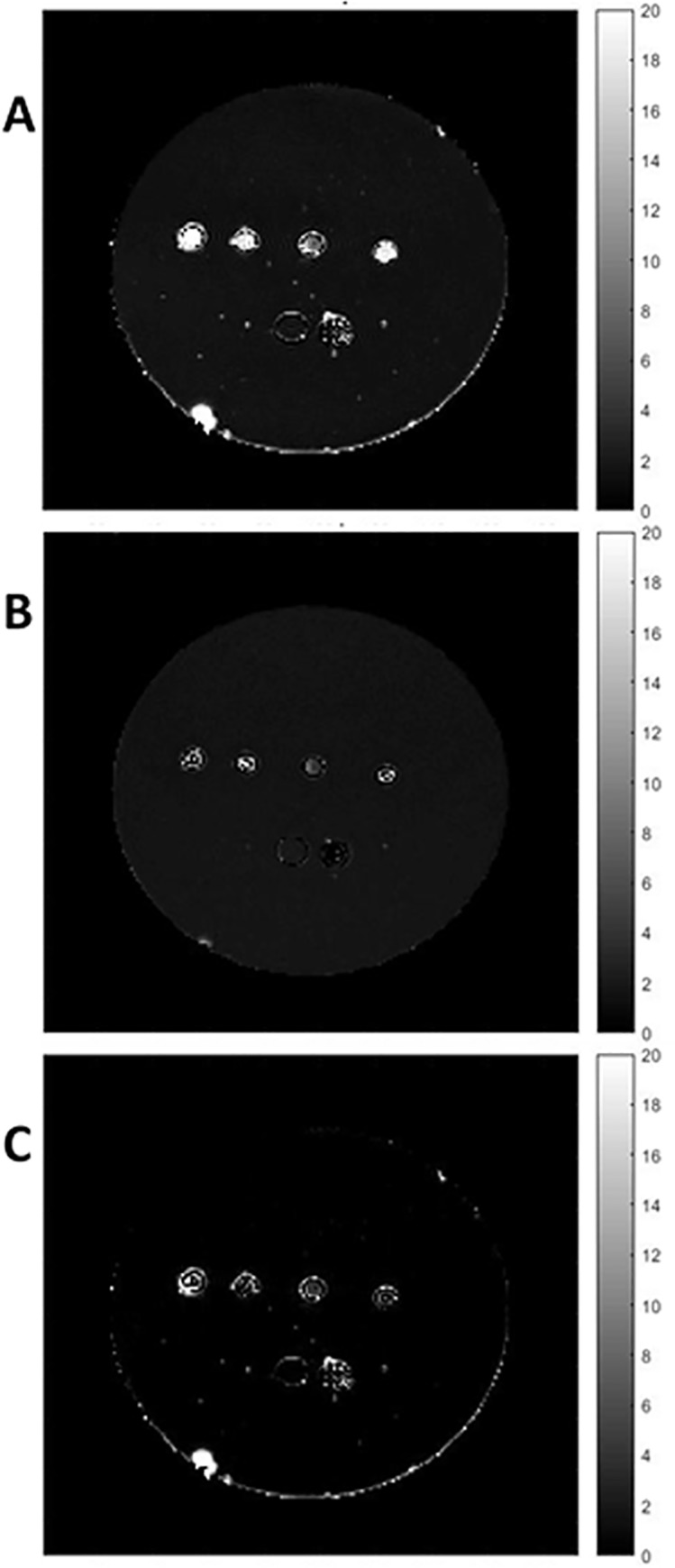
Transverse relaxation rate mapping. Representative maps are shown for R2 (A), R2* (B) and R2′ (C). The first two maps were obtained using voxel by voxel curve fitting with an exponential decay function and the R2′ map was obtained by subtraction (R2* ‒ R2). The units of the scale bar are S^-1^. Images show sample wells in the phantom, in cross section. From left to right in the top row are M 24h-Fe, P 24h-Fe, P and P+Fe. Along the bottom row, from left to right, there is a well filled with 4% gelatin and a polystyrene marker for reference. Maps are provided for display only; relaxation rates (Fig 8) were determined as outlined in Methods.

**Fig 8:**
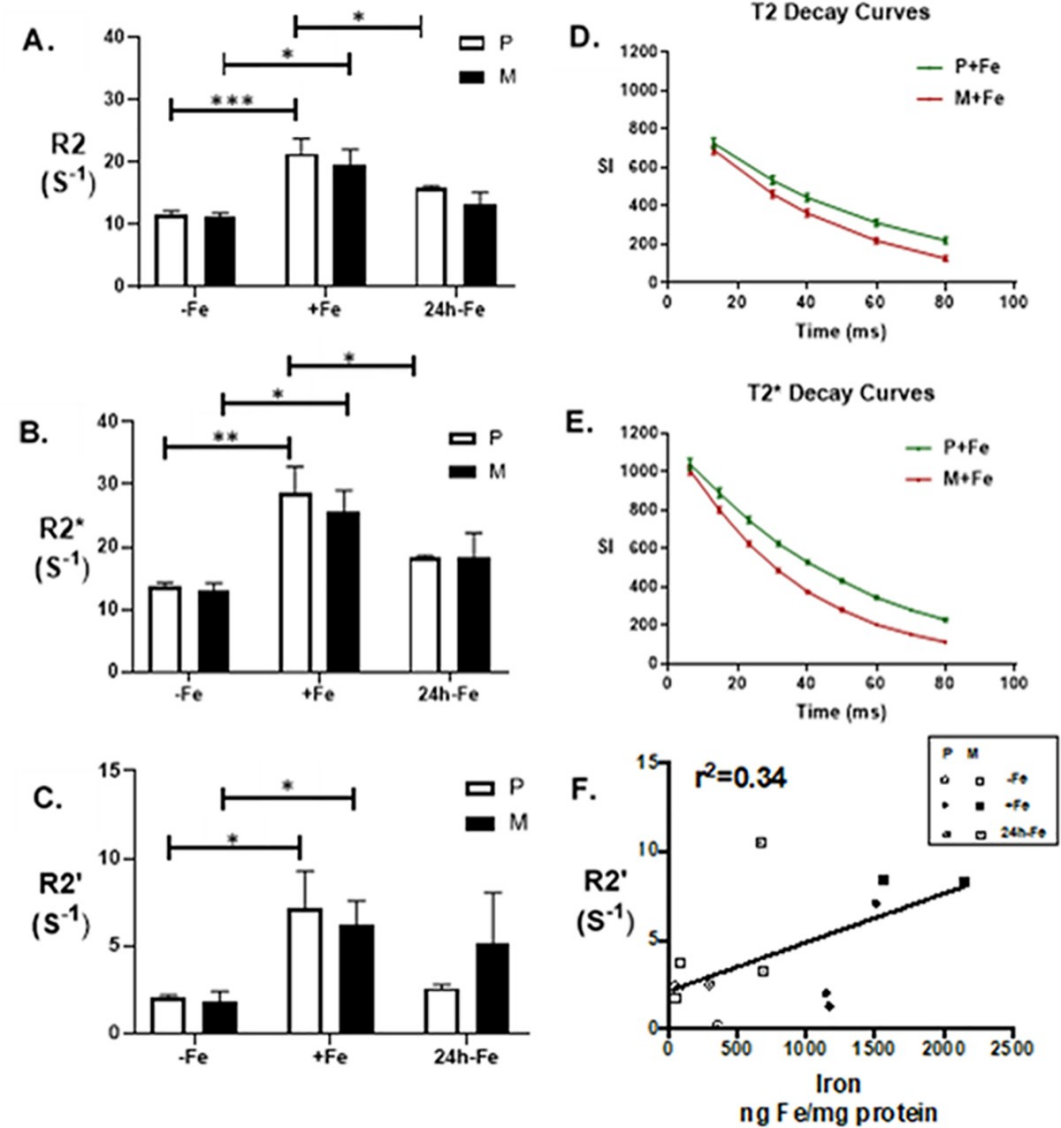
Influence of iron supplementation and MagA expression on transverse relaxivity in P19 cells. Parental cells (P, white bars) and MagA-expressing cells (M, black bars) were cultured in the absence of iron supplementation (-Fe), in medium containing 250 μM ferric nitrate for 7 days (+Fe) and after withdrawal of iron from +Fe samples for 24 hours (24h-Fe). Cells were mounted in gelatin phantoms and scanned at 3T as previously described [6]. The irreversible component of transverse relaxation rate (R2, A) showed no significant difference between cells types within a given culture condition; however, R2 increased significantly upon iron supplementation (+Fe). At 24h-Fe, only the parental cell type showed a significant decrease in R2. The total transverse relaxation rate (R2*, B) also showed no significant difference between cell types within a given culture condition; a significant increase upon iron supplementation (+Fe); and a significant decrease at 24h-Fe only in the parental cell type. Comparison of T2 (D) and T2* (E) relaxation curves over 80 ms revealed a greater difference in signal decay between parental (green) and MagA-expressing cells (red) for T2* acquisitions compared to T2. The reversible component of transverse relaxation rate (R2′, C) represents the difference between R2* and R2 and displayed the same significant increase in each cell type upon iron supplementation. However, neither parental nor MagA-expressing cells displayed significant decreases in R2′ at 24h-Fe. There was a moderate correlation between elemental iron content and R2′ (F). Error bars are ± SEM (* p < 0.05; ** p < 0.01; *** p < 0.001); for parental samples, n = 7–9; for MagA-expressing samples, n = 3–4.

Previous studies showed that total cellular iron content is significantly correlated to the reversible transverse relaxation rate, R2′ [14]. In the present study, transverse relaxation rates, R2, R2* and R2ʹ, were all notably different in both parental and MagA-expressing P19 cells (Fig 7). For each relaxation rate, significant main effects for the iron condition were found using two-way ANOVA (Fig 8, p < 0.001). Bar charts show the mean values of transverse relaxation rates for parental and MagA-expressing P19 cells cultured under 3 different conditions: -Fe, +Fe and 24h-Fe. Significant differences in R2 are observed in both parental and MagA-expressing P19 cells for continuously iron-supplemented and non-supplemented conditions (Fig 8A, P-Fe vs. P+Fe, p < 0.001 and M-Fe vs. M+Fe, p < 0.05). Upon withdrawal of iron supplement for 24 hours (24h-Fe), only parental P19 cells exhibit a significant decrease in R2 (p < 0.05). The same pattern is observed for R2* measurements (Fig 8B).

Comparing the signal decay curves over 80 ms (Fig 8D and 8E), R2* (1/T2*) shows a higher rate (with respect to TE) for the gradient echo signal compared to the spin-echo signal (*i*.*e*., R2* > R2). This is consistent with a previous study in MDA-MB-435 cells [6]. R2* represents the total transverse relaxation rate and includes both the irreversible R2 component and the reversible R2′ component. In Fig 8E, R2* decay shows a greater difference between parental and MagA-expressing cells than the R2 component alone. Examination of this difference (R2* ‒ R2 = R2′; Fig 8C) shows that R2′ is comparable between parental and MagA-expressing P19 cells cultured in either the absence (-Fe) or presence (+Fe) of iron supplementation (n = 4–9). In addition, both cell types showed significantly higher R2ʹ between these two iron conditions (P-Fe vs. P+Fe and M-Fe vs. M+Fe, p < 0.05). After at least 1 week of continuous iron supplementation, parental and MagA-expressing P19 cells were returned to culture for a further 24 hours in non-supplemented medium (24h-Fe). In parental cells, the apparent decrease in R2′ nevertheless fails to meet statistical significance (+Fe vs. 24h-Fe, p = 0.055) despite approaching baseline values (2.00 ± 0.06 s^-1^ for -Fe, n = 7 and 2.48 ± 0.07 s^-1^ for 24h-Fe, n = 8). This result may be partially explained by the moderate correlation between total cellular iron content and R2′ in the P19 cell system (Fig 8F).

## Discussion

This report describes the successful expression of *magA*, a gene from *Magnetospirillum magneticum* species AMB-1, in undifferentiated P19 mouse embryonal carcinoma cells and generation of a stable MagA-expressing clonal cell line. Our study reports the cellular location of MagA using immunocytochemistry, the response of MagA-expressing cells to extracellular iron supplementation using mass spectrometry and their magnetic properties using MR relaxation rates. A comparison to parental P19 cells reveals (1) pronounced iron uptake and export functions not previously described in this cell line; (2) the influence of MagA on mammalian iron export; and (3) the degree to which MagA expression provides MR contrast in an iron-exporting cell type.

### Protein analysis

MagA expression was detected using an HA tag fused to the C-terminus and a commercial antibody. A Western blot of MagA-HA-expressing P19 cells identified a highly expressing clonal cell line. The apparent M.W. of MagA-HA was approximately 35 KDa in agreement with a recent report using HA-tagged MagA to study the MR signal in a mouse embryonic stem cell line [4]. Although the predicted M.W. of MagA-HA is approximately 47 KDa, no post-translational processing of the N-terminal has been reported to date.

To localize the expression of MagA in P19 cells, ICC was performed. In bacteria, MagA is a putative iron transport protein localized in the membrane compartment [15, 16]. In mammalian P19 cells, HA-tagged protein was predominantly immuno-stained within the intracellular compartment of MagA-expressing cells. The possibility of plasma membrane localization was examined using WGA, an approximately 36 kDa carbohydrate-binding protein with an affinity for sialic acid and N-acetylglucosaminyl sugar residues, which are predominately expressed on the plasma membrane of mammalian cells [17]. Immunocytochemical analysis showed that while MagA and WGA co-localize at the plasma membrane of P19 cells, the majority of MagA immunostaining resided within the intracellular compartment(s).

To investigate intracellular localization of MagA, we used an antibody specific for p115, a Golgi Apparatus associated protein. In mammalian cells, proteins are synthesized in the endoplasmic reticulum and transported to the Golgi complex for processing and sorting [18]. While these cargo proteins may encompass a wide spectrum of properties, the dynamic nature of the Golgi network provides several routes by which cargo may be shuttled to their final destination [19]. The adapter protein p115 is required for vesicle transport from the cis to the medial compartments [20, 21]. Co-localization of MagA and p115 implies that overexpressed protein may be accumulating in the Golgi vesicle(s) responsible for organizing membrane-associated protein within the cell. These results are consistent with published [5, 16] and unpublished data indicating that MagA is a membrane protein. In addition, since all proteins do not traverse the Golgi at the same speed [19], it is perhaps not surprising to observe substantial expression of a foreign gene, like MagA, within the cis-Golgi. As a multi-pass transmembrane protein, MagA may be subject to regulatory pathways that involve lipid. Newly recognized roles of the Golgi in cellular metabolism [22] may also predispose MagA to as yet unrecognized intracellular processing, such as glycosylation. In an aggressively mitotic cancer cell like P19, rapid cycles of Golgi ribbon disassembly and reassembly with each cell division is also postulated to regulate cellular processes [23]. Further studies are needed to properly understand the intracellular localization of MagA in mammalian cells. The possibility of iron accumulation in intracellular vesicles of MagA-expressing cells [7] is not without precedent. For example, dopamine neurovesicles accumulate iron and are disrupted in Parkinson’s disease [24].

### Iron analysis

Mammalian cells display elaborate regulation of iron uptake, storage, export and distribution of intracellular iron [25]. It is a constituent of such important proteins as hemoglobin, cytochromes, oxygenases, flavoproteins, and redoxins [26]. For iron uptake, transferrin is an important extracellular antioxidant that binds iron tightly under physiological conditions, providing very little unbound iron for the production of free radicals. The delivery of iron to cells involves receptor-mediated uptake of transferrin-bound iron [27]. Moreover, by controlling the expression of transferrin receptor, through the interaction of iron binding proteins with iron response elements, mammalian cells regulate the amount of iron they import from the extracellular environment. This form of transferrin receptor-mediated iron uptake is present in most cell types [28]. In P19 cells, we showed that transferrin receptor expression and iron uptake activity prevail no matter the duration of exposure to extracellular iron or a 10-fold variation in its concentration. In contrast, Ferroportin 1 (Fpn1) is the only iron exporter identified to date [25, 29–31] and its expression is largely restricted to enterocytes, macrophages, hepatocytes [32], placental syncytiotrophoblasts [33] and breast epithelium [34]. Once Fe(II) is exported across the basal membrane of the cell by Fpn1, the iron is oxidized by hephaestin, a multi-copper oxidase that interacts with plasma transferrin [35]. A subsequent study has implicated hepcidin regulation of P19 iron export [36].

Thus, iron and its careful regulation are of crucial importance to living cells. In cultured P19 cells, total cellular iron content increases dramatically in response to an extracellular iron supplement, regardless of MagA expression. Continuous culture in the presence of iron-supplemented medium results in a significant 25-fold increase (p < 0.001) in total cellular iron content. However, in parental cells this also decreased abruptly with time when iron supplementation was withdrawn. Within two hours, the total cellular iron content decreased by 40% to 630 ± 123 ng iron/mg protein. By 24 hours, 70% of the iron incorporated by +Fe treatment had dissipated (p < 0.05).

MagA-expressing P19 cells exhibited a 33-fold increase in total cellular iron content, significantly above the uptake of iron displayed by the parental cell type (p < 0.05) after week long culture in iron-supplemented medium. When extracellular supplementation was withdrawn, iron export persisted in MagA-expressing cells with a 40% decrease in total iron at 2h-Fe, in parallel with the parental cell type, but with only a 50% decrease over 24 hours. In MagA-expressing cells, total cellular iron content was more than 2-fold greater than the parental control even after withdrawal of iron supplement for 24 h (p < 0.05). The iron export function of P19 cells is unexpected and has been confirmed by Western blots identifying ferroportin protein expression [14]. The previously unrecognized iron handling abilities of P19 cells are similar to what is reported for both mouse [37] and human [38] M2 macrophages. In the murine model, macrophages were cultured from bone marrow-derived precursors and differentiated into inflammatory (M1) or alternative (M2) phenotypes. The iron recycling ability of M2 cells that exhibit both high iron import and export functions is distinct from the iron storage capacity exhibited by M1 cells, underlining the potential for distinguishing these changes in iron handling noninvasively using MRI. Moreover, the ability of MagA expression to retain iron in P19 cells indicates the potential of MagA activity to modulate intrinsic iron export function and may have therapeutic applications in the management of iron overload diseases [39].

### MRI analysis

While the exact mechanism of MagA function in bacterial and mammalian cells has not been fully characterized, the results in P19 agree with previous studies indicating that MagA expression increases iron incorporation in mammalian cells. With measurement of relaxation rates, we explored the potential for contrast enhancement in P19 cells by the expression of MagA. Longitudinal relaxation rates were influenced very little by the striking increase in cellular iron and this is consistent with other publications [6, 40]. On the other hand, the transverse relaxation rates (R2* and R2) were strongly affected by iron supplementation in both parental and MagA-expressing P19 cells.

Compared to unsupplemented P19 cells, either parental or MagA-expressing, the transverse relaxation rates (R2* and R2) are significantly higher in iron-supplemented cells. However, there is no significant difference in these relaxation rates between MagA-expressing and parental P19 cells cultured continuously in the presence of iron-supplemented medium. This is partially consistent with ICP-MS results, which show an approximately 33-fold (M+Fe) and 25-fold (P+Fe) rise in total cellular iron content in iron-supplemented MagA-expressing cells and the respective P19 control. However, these data are not consistent with previous studies [4–7]. In other cell types, like MDA-MB-435, which downregulate transferrin receptor expression in response to iron supplementation, there is little or no increase in cellular iron content or relaxation rate in the absence of MagA expression [6]. Results in P19 again suggest that the parental cell type possesses high iron import activity. We note that, while the activity of MagA permits 40–50% greater cellular iron content than the P19 control, transverse relaxivity cannot distinguish this increase using the cell phantom and (clinical) MR scanning parameters employed in this study.

The detection of iron export activity in P19 cells prompted an investigation of relaxation rates in samples collected 24 hours after the removal of iron supplement. The decrease in R2 and R2* measurements at this time point in the parental cell type reflects an approximately 3-fold decrease in total cellular iron content relative to +Fe samples. Smaller decreases as seen in MagA-expressing P19 cells were not readily distinguished by MRI. We examined the iron specificity of R2′ measurements for the potential to discern smaller differences in cellular iron content (Fig 6C). R2′ was strongly affected by iron supplementation in both parental and MagA-expressing P19 cells (p < 0.05) but did not reveal significant changes in MR contrast 24 hours after the withdrawal of extracellular iron, even in parental P19 cells (p = 0.055). In MDA-MB-435 [6], the correlation between R2′ and total cellular iron content for MagA-expressing cells is robust (r = 0.96), with a low y-intercept indicating better iron-related specificity than R2 [14]. In P19 cells, the correlation between R2′ and total cellular iron content for parental and MagA-expressing P19 cells is only moderately strong (Fig 8F, r = 0.58). These data suggest that the iron recycling phenotype, reflecting flux of iron in and out of the cell rather than iron storage, may dominate the signal attributed to transverse relaxivity and partly explain the poorer correlation. Although elemental iron content is greater in MagA-expressing cells, the form of that iron is not reported by ICP-MS. In cells exhibiting high iron import and iron export function, and a lower capacity for iron storage, as detected in P19 cells under -Fe conditions, paramagnetic forms of protein-bound iron are expected to be associated with relatively more transferrin receptor and ferroportin than ferritin. The dynamic nature of iron import and export, including transitions between ferric and ferrous iron, may also contribute to variability in the MR signal [41]. In addition, while the larger labile iron pool reported in iron recycling cells [37] is not expected to contribute much to the paramagnetic signal [42], MagA expression may draw iron away from export by ferroportin and potentially decrease a relatively MR-invisible labile iron pool. In this case, any rudimentary magnetosome-like particle resulting from MagA-derived contrast in P19 cells may be too small and/or lacking the optimal paramagnetic form for reliable MR detection. In the future, this may be improved by the expression of a combination of magnetosome genes [39]. Further experiments are warranted to fully characterize the iron export activity in parental and MagA-expressing P19 cells. In the future, the rate of iron export may potentially be used to calibrate the flux in cellular iron content and its relationship to MRI measurements.

## Conclusion

We examined the expression and function of MagA in undifferentiated P19 cells to further extend the development of gene-based iron labelling for MRI. Relaxation rates were investigated in the context of total cellular iron content in both parental and MagA-expressing cells cultured under various conditions of iron supplementation (-Fe, +Fe and 24h-Fe). The results reveal new features of P19 iron biochemistry and demonstrate the potential for MagA to retain iron in P19 cells. Western blots and ICC demonstrated the expression and membrane localization of MagA-HA in P19 cells. Extracellular iron supplementation of cultured cells significantly increased the total iron content in both parental and MagA-expressing P19 cells, resulting in an increase in transverse relaxation rates. Withdrawal of iron supplementation revealed substantial iron export activity in parental P19 cells. MagA expression attenuated this iron export function, permitting the cell to retain iron and MR contrast for a longer period of time. This is the first report of the influence of MagA expression on an iron exporting cell type. While this feature of P19 iron metabolism diminishes the magnitude of the cellular signal from MagA-derived MR contrast, it also highlights a potentially new use of MagA in the regulation of iron export as well as the possibility of tracking iron-exporting cells without the need for additional contrast.

## Supporting information

S1 Fig Western blot confirming MagA-expression in transfected but not untransfected P19 cells. Lanes 1 and 2 contain protein extracted from untransfected parental P19 cells while lanes 3–6 contain protein from MagA-HA-expressing cells. The blot was probed with primary antibody against HA, as described in Methods. No HA-tagged protein was detected in untransfected P19 cells (lanes 1 and 2). Although several clones of MagA-HA-expressing cells were detected (lanes 3–6), none of these were selected for the reported experiments. Approximate M.W. is indicated on the left.
(TIF)

S2 Fig Time course of iron export in parental and MagA-expressing P19 cells. Parental P19 cells (P, white circles) and those expressing MagA (M, black squares) were cultured for at least 7 days in the presence (+Fe) of iron supplementation (250 μM ferric nitrate∕medium) prior to withdrawal of iron supplement and culture for an additional 1, 2 and 24 hours. Total cellular iron content was analyzed by ICP-MS and normalized to total cellular protein. After iron supplementation, iron content in MagA-expressing cells was significantly higher than in untransfected cells (red asterisk at time 0) and remained higher following iron withdrawal for 2 to 24 hours (red asterisks). Cellular iron content decreased significantly in parental cells after 24h of iron withdrawal (blue asterisk) but not in MagA-expressing cells. Error bars are ± SEM (* p < 0.05). For +Fe, n = 5–7; for all other samples, n = 3.
(TIF)

## References

[1] Massoud T, Gambhir S. Molecular imaging in living subjects: seeing fundamental biological processes in a new light. Genes Dev. 2003;17:545–80. 10.1101/gad.1047403 12629038

[2] James M, Gambhir S. A molecular imaging primer: modalities, imaging agents, and applications. Physiol Rev. 2012;92:897–965. 10.1152/physrev.00049.2010 22535898

[3] Geraldes C, Laurent S. Classification and basic properties of contrast agents for magnetic resonance imaging. Contrast Media MoI Imaging. 2009;4:1–23. 19156706 10.1002/cmmi.265

[4] Cho I, Moran S, Paudyal R, Piotrowska-Nitsche K, Cheng P, Zhang X, et al Longitudinal monitoring of stem cell grafts in vivo using magnetic resonance imaging with inducible maga as a genetic reporter. Theranostics. 2014;4(10):972–89. 10.7150/thno.9436 25161700 PMC4143941

[5] Goldhawk D, Lemaire C, McCreary C, McGirr R, Dhanvantari S, Thompson R, et al Magnetic resonance imaging of cells overexpressing magA, an endogenous contrast agent for live cell imaging. Mol Imaging. 2009;8(3):129–39. 19723470

[6] Sengupta A, Quiaoit K, Thompson R, Prato F, Gelman N, Goldhawk D. Biophysical features of MagA expression in mammalian cells: implications for MRI contrast. Front Microbiol. 2014;5:29 10.3389/fmicb.2014.00029 24550900 PMC3913841

[7] Zurkiya O, Chan A, Hu X. MagA is sufficient for producing magnetic nanoparticles in mammalian cells, making it an MRI reporter. Magn Reson Med. 2008;59(6):1225–31. 10.1002/mrm.21606 18506784 PMC4416408

[8] Rohani R, Figueredo R, Bureau Y, Koropatnick J, Foster P, Thompson R, et al Imaging tumor growth non-invasively using expression of MagA or modified ferritin subunits to augment intracellular contrast for repetitive MRI. Mol Imaging Biol. 2014;16:63–73. 10.1007/s11307-013-0661-8 23836502

[9] Anazodo U, Finger E, Kwan B, Pavlosky W, Warrington J, Günthere M, et al Using simultaneous PET/MRI to compare the accuracy of diagnosing frontotemporal dementia by arterial spin labelling MRI and FDG-PET. NeuroImage Clinical. 2018;17:405–14. 10.1016/j.nicl.2017.10.033 29159053 PMC5683801

[10] Ssali T, Anazodo U, Thiessen J, Prato F, St Lawrence K. A Non-invasive method for quantifying cerebral blood flow by hybrid PET/MR. J Nuc Med. 2018;59:1329–34. 10.2967/jnumed.117.203414 29523628

[11] Genove G, DeMarco U, Xu H, Goins W, Ahrens E. A new transgene reporter for in vivo magnetic resonance imaging. Nat Med. 2005;11:450–4. 10.1038/nm1208 15778721

[12] Smith P, Krohn R, Hermanson G, Mallia A, Gartner F, Provenzano M, et al Measurement of protein using bicinchoninic acid. Anal Biochem. 1985;150:76–85. 3843705 10.1016/0003-2697(85)90442-7

[13] Gelman N, Gorell J, Barker P, Savage R, Spickler E, Windham J, et al MR imaging of human brain at 3.0 T: preliminary report on transverse relaxation rates and relation to estimated iron content. Radiology. 1999;210:759–67. 10.1148/radiology.210.3.r99fe41759 10207479

[14] Goldhawk D, Gelman N, Sengupta A, Prato F. The interface between iron metabolism and gene-based iron contrast for MRI. Magnetic Resonance Insights. 2015;8(S1):9–14. 26483608 10.4137/MRI.S23555 PMC4597585

[15] Nakamura C, Burgess JG, Sode K, Matsunaga T. An iron-regulated gene, *magA*, encoding an iron transport protein of *Magnetospirillum* sp. Strain AMB-1. J Biol Chem. 1995;270:28392–6. 10.1074/jbc.270.47.28392 7499342

[16] Nakamura C, Kikuchi T, Burgess JG, Matsunaga T. Iron-regulated expression and membrane localization of the MagA protein in *Magnetospirillum* sp. Strain AMB-1. J Biochem. 1995;118:23–7. 10.1093/oxfordjournals.jbchem.a124884 8537318

[17] Wright C. Structural comparison of the two distinct sugar binding sites in wheat germ agglutinin isolectin II. J Mol Biol. 1984;178:91–104. 6548265 10.1016/0022-2836(84)90232-8

[18] Huang S, Wang Y. Golgi structure formation, function and post-translational modifications in mammalian cells. F1000Research. 2018;6:e2050. 10.12688/f1000research.11900.1 PMC5710388 29225785

[19] Boncompain G, Weigel A. Transport and sorting in the Golgi complex: multiple mechanisms sort diverse cargo. Curr Opin Cell Biol. 2018;50:94–101. 10.1016/j.ceb.2018.03.002 29567348

[20] Barroso M, Nelson D, Sztul E. Transcytosis-associated protein (TAP)/p115 is a general fusion factor required for binding of vesicles to acceptor membranes. Proc Natl Acad Sci USA. 1995;92:527–31. 10.1073/pnas.92.2.527 7831324 PMC42774

[21] Waters M, Clary D, Rothman J. A novel 115-kD peripheral membrane protein is required for intercisternal transport in the Golgi stack. J Cell Biol. 1992;118:1015–26. 10.1083/jcb.118.5.1015 1512287 PMC2289595

[22] Gosavi P, Gleeson P. The Function of the Golgi Ribbon Structure–An Enduring Mystery Unfolds! BioEssays. 2017;39:e1700063. 10.1002/bies.201700063 28984991

[23] Makhoul C, Gosavi P, Gleeson P. The Golgi architecture and cell sensing. Biochem Soc Trans. 2018;46:1063–72. 10.1042/BST20180323 30242119

[24] Ortega R, Cloetens P, Deve`s G, Carmona A, Bohic S. Iron Storage within Dopamine Neurovesicles Revealed by Chemical Nano-Imaging. PLoS ONE. 2007;2:e925 10.1371/journal.pone.0000925 17895967 PMC1976597

[25] Hentze M, Muckenthaler M, Andrews N. Balancing acts: molecular control of mammalian iron metabolism. Cell. 2004;117:285–97. 15109490 10.1016/s0092-8674(04)00343-5

[26] MacKenzie E, Iwasaki K, Tsuji Y. Intracellular iron transport and storage: from molecular mechanisms to health implications. Antioxid Redox Signal. 2008;10:997–1030. 10.1089/ars.2007.1893 18327971 PMC2932529

[27] Cheng Y, Zak O, Aisen P, Harrison S, Walz T. Structure of the human transferrin receptor-transferrin complex. Cell. 2008;116:565–76. 10.1016/s0092-8674(04)00130-8 14980223

[28] Hentze M, Muckenthaler M, Galy B, Camaschella C. Two to tango: regulation of mammalian iron metabolism. Cell. 2010;142:24–38. 10.1016/j.cell.2010.06.028 20603012

[29] Abboud S, Haile D. A novel mammalian iron-regulated protein involved in intracellular iron metabolism. J Biol Chem. 2000;275:19906–12. 10.1074/jbc.M000713200 10747949

[30] Donovan A, Zhou Y, Shepard J, Pratt S, Moynihan J, Paw B, et al Positional cloning of zebrafish ferroportin 1 identifies a conserved vertebrate iron exporter. Nature. 2000;403:776–81. 10.1038/35001596 10693807

[31] McKie A, Marciani P, Rolfs A, Brennan K, Wehr K, Barrow D, et al A novel duodenal iron-regulator transporter, IREG1, implicated in the basolateral transfer of iron to the circulation. Mol Cell. 2000;5:299–309. 10882071 10.1016/s1097-2765(00)80425-6

[32] Ganz T, Nemeth E. Hepcidin and iron homeostasis. Biochim Biophys Acta. 2012;1823:1434–43. 10.1016/j.bbamcr.2012.01.014 22306005 PMC4048856

[33] McKie A, Barlow D. The SLC40 basolateral iron transporter family (IREG1/ferroportin/MTP1). Pflugers Arch—Eur J Physiol. 2004;447:801–6. 12836025 10.1007/s00424-003-1102-3

[34] Pinnix Z, Miller L, Wang W, D’Agostino R Jr., Kute T, Willingham M, et al Ferroportin and iron regulation in breast cancer progression and prognosis. Sci Transl Med. 2010;2:43ra56 10.1126/scisignal.3001127 20686179 PMC3734848

[35] Vulpe C, Kuo Y-M, Murphy T, Cowley L, Askwith C, Libina N, et al Hephaestin, a ceruloplasmin homologue implicated in intestinal iron transport, is defective in the sla mouse. Nat Genet. 1999;21:195–9. 10.1038/5979 9988272

[36] Alizadeh Pourbouyeh K. Hepcidin-mediated Iron Regulation in P19 Cells is Detectable by MRI. Western University, London, Canada, Scholarship@Western Electronic Thesis and Dissertation Repository.2018.

[37] Corna G, Campana L, Pignatti E, Castiglioni A, Tagliafico E, Bosurgi L, et al Polarization dictates iron handling by inflammatory and alternatively activated macrophages. Haematologica. 2010;95:1814–22. 10.3324/haematol.2010.023879 20511666 PMC2966902

[38] Recalcati S, Locati M, Marini A, Santambrogio P, Zaninotto F, De Pizzol M, et al Differential regulation of iron homeostasis during human macrophage polarized activation. Eur J Immunol. 2010;40:824–35. 10.1002/eji.200939889 20039303

[39] Goldhawk D, Gelman N, Thompson R, Prato F. Forming magnetosome-like nanoparticles in mammalian cells for molecular MRI In: Bulte J, Modo M, editors. Design and Applications of Nanoparticles in Biomedical Imaging. Switzerland: Springer International Publishing; 2017 p. 187–203.

[40] Na H, Song I, Hyeon T. Inorganic nanoparticles for MRI contrast agents. Adv Mater. 2009;21:2133–48.

[41] Dietrich O, Levin J, Ahmadi S-A, Plate A, Reiser M, Bötzel K, et al MR imaging differentiation of Fe2+ and Fe3+ based on relaxation and magnetic susceptibility properties. Neuroradiology. 2017;59:403–9. 10.1007/s00234-017-1813-3 28324122

[42] Tubb B. Tissue Iron Deposition: MRI Quantitation In Clinical Trials. Intrinsic Imaging. 2016;6(6):1–5.

