# Supplementary figures and images for: MagA expression attenuates iron export activity in undifferentiated multipotent P19 cells
Source: PLoS One. 2019 Jun 6;14(6):e0217842. doi: 10.1371/journal.pone.0217842 (PMC6553743; doi:10.1371/journal.pone.0217842)

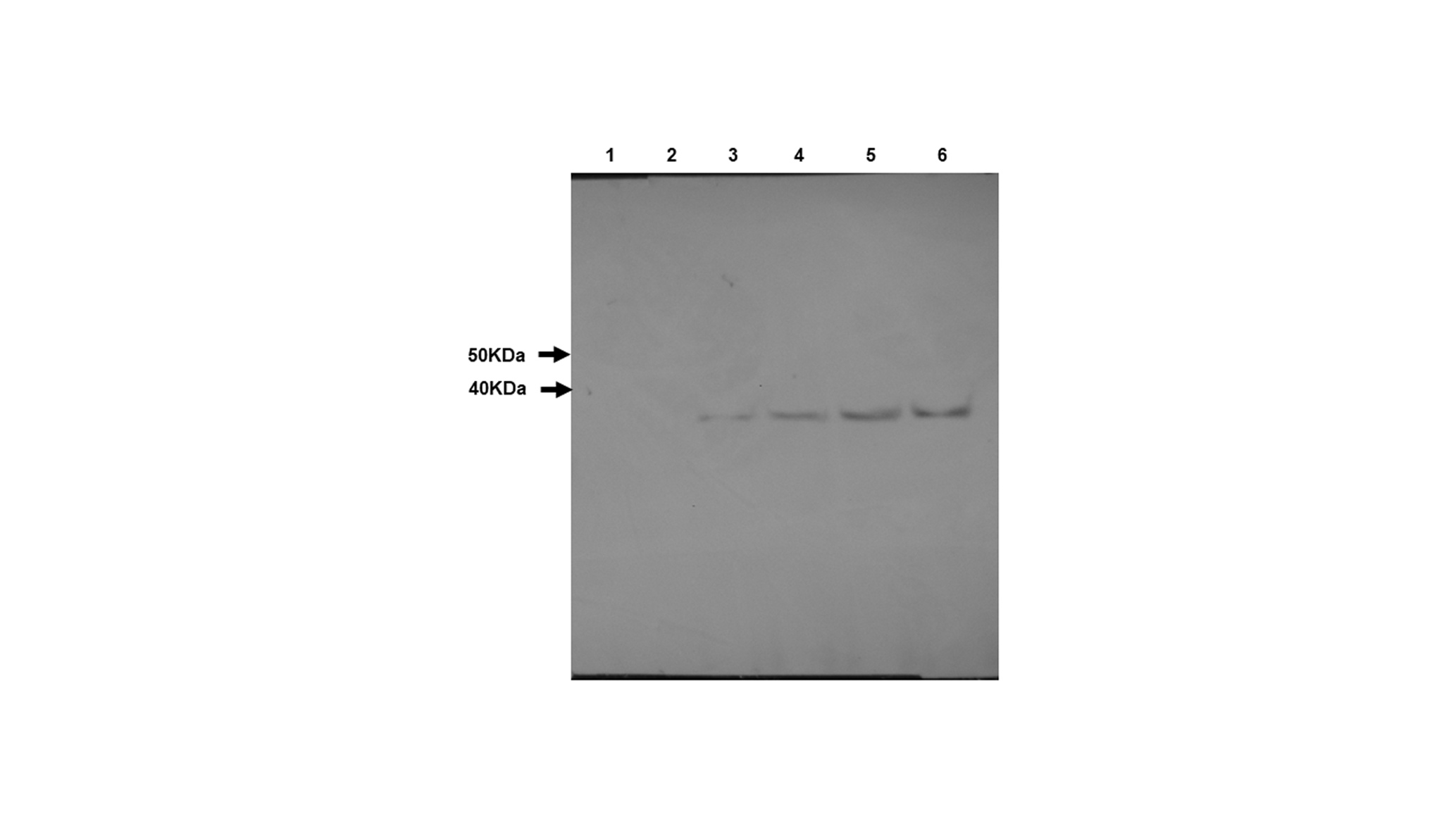

Supplement: S1 Fig — Lanes 1 and 2 contain protein extracted from untransfected parental P19 cells while lanes 3–6 contain protein from MagA-HA-expressing cells. The blot was probed with primary antibody against HA, as described in Methods. No HA-tagged protein was detected in untransfected P19 cells (lanes 1 and 2). Although several clones of MagA-HA-expressing cells were detected (lanes 3–6), none of these were selected for the reported experiments. Approximate M.W. is indicated on the left. (TIF) [file pone.0217842.s001.tif]

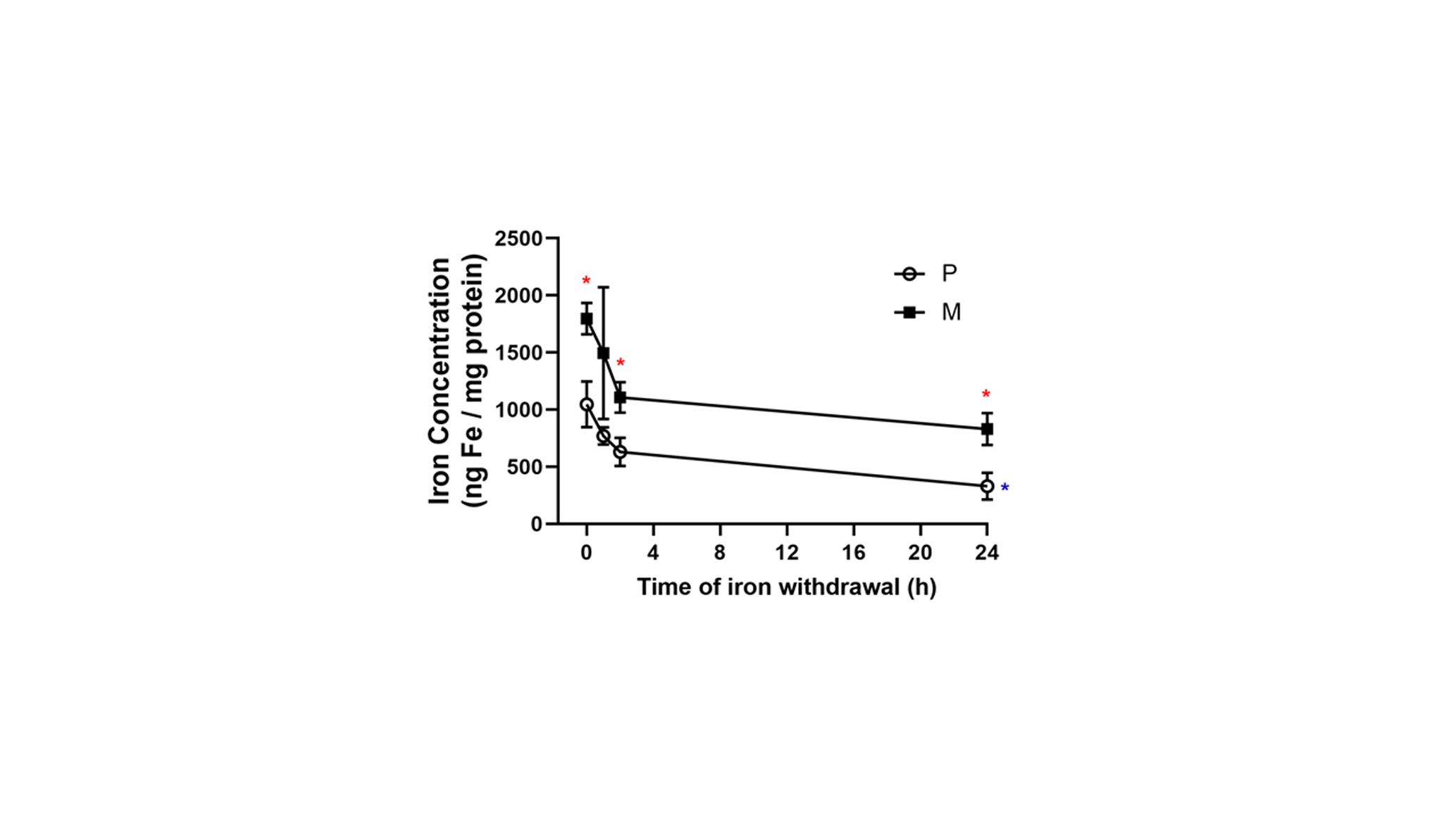

Supplement: S2 Fig — Parental P19 cells (P, white circles) and those expressing MagA (M, black squares) were cultured for at least 7 days in the presence (+Fe) of iron supplementation (250 μM ferric nitrate∕medium) prior to withdrawal of iron supplement and culture for an additional 1, 2 and 24 hours. Total cellular iron content was analyzed by ICP-MS and normalized to total cellular protein. After iron supplementation, iron content in MagA-expressing cells was significantly higher than in untransfected cells (red asterisk at time 0) and remained higher following iron withdrawal for 2 to 24 hours (red asterisks). Cellular iron content decreased significantly in parental cells after 24h of iron withdrawal (blue asterisk) but not in MagA-expressing cells. Error bars are ± SEM (* p < 0.05). For +Fe, n = 5–7; for all other samples, n = 3. (TIF) [file pone.0217842.s002.tif]
